# Age and gender differences of psychogenic fever: a review of the Japanese literature

**DOI:** 10.1186/1751-0759-1-11

**Published:** 2007-05-19

**Authors:** Takakazu Oka, Kae Oka

**Affiliations:** 1 Psychosomatic Medicine Division, Department of Neurology, University of Occupational and Environmental Health, Japan Iseigaoka 1-1, Yahatanishi-ku, Kitakyushu, 807-8555, Japan; 2 Pediatrics Department, Faculty of Medicine, Kurume University Asahimachi 67, Kurume, 830-0011, Japan

## Abstract

**Background:**

Psychogenic fever is one of the most common psychosomatic diseases. Patients with psychogenic fever have acute or persistent body temperature above normal range in psychologically stressful situations. In spite of numerous case reports on psychogenic fever, there are few epidemiological studies. Therefore, our goal was to investigate the age distribution and gender differences of psychogenic fever in Japan.

**Methods:**

To achieve this goal, we searched Medline and Ichushi WEB, a Japanese medical database, and added other publications that were not included in these databases. Thus, we reviewed 195 Japanese cases of psychogenic fever published in 62 papers.

**Results:**

Psychogenic fever patients ranged from 3 to 56 years old, with the highest number of cases occurring in 13 year-olds in both sexes. The male: female ratio of 1: 1.19 suggested a slight predominance of female cases. Psychogenic fever accounted for 18% of fever cases of unknown origin in children and 2–6% of the psychosomatic diseases of pediatric patients. Patients with psychogenic fever were not only found in pediatrics departments, but also in psychosomatic medicine, psychiatry, internal medicine, anesthesiology, dentistry, and obstetrics/gynecology departments.

**Conclusion:**

The age of psychogenic fever patients ranged from 3 to 56 years old and the male: female ratio was 1:1.19. Psychogenic fever is seen especially in adolescence in Japan.

## Background

Psychogenic fever is one of the most common psychosomatic diseases; physical diseases affected by psychosocial factors. Psychogenic fever is diagnosed when (1) there is no organic disease that accounts for the fever and (2) the fever develops in a psychologically stressful situation or (3) emotionally stressful stimuli induce acute or persistent increases in core temperature (Tc) above the upper limit of normal body temperature (37°C). Although numerous case reports on psychogenic fever have been published, the mechanisms by which psychological stress increases core temperature are not fully understood, and epidemiological studies are limited. Therefore, our primary objective was to investigate the age distribution and gender differences of psychogenic fever in Japan by reviewing the published literature.

## Methods

To achieve this goal, on January 15^th^, 2007 using the key words "psychogenic fever" we performed electronic searches of Medline (1960–2006) and Ichushi WEB (1983 (the beginning of the service)-2006), a database of the NPO Japan Medical Abstract Society. The search identified 61 publications that included original papers, case reports, review articles, abstracts, or proceedings. Because the aim of this study was to determine the age distribution and gender differences of Japanese patients with psychogenic fever, we included abstracts and proceedings if the age and gender of the patients were described. However, the following were excluded from this study: (1) abstracts and proceedings that were subsequently published as original articles or case reports; (2) papers that did not distinguish psychogenic fever from factitious fever; (3) review articles that did not refer to cases; (4) papers that exclusively described animal studies; and (5) papers in which the involvement of psychosocial factors on the development of fever was unclear. Thus, 23 papers were excluded. We additionally included 24 papers that the reviewed papers had cited or that we personally identified but that were not included in Medline or Ichushi WEB. Thus, in total we reviewed 62 papers [1-62].

### Statistic analysis

Data are shown as the mean ± standard deviation (SD). Student's *t*-test for unmatched data and Kolmogorov-Smirnov test were applied to analyze statistical differences between groups.

## Results

The papers that we reviewed were segregated into two types. ''Type 1'' includes those in which we could identify the age and sex of each patient. ''Type 2'' includes papers in which age and gender were described in groups, such as five patients (three male and two female) between 8–10 years old. Of the 195 cases identified, 135 were type 1 and 60 were type 2. If the paper described the patients as being in the 1^st^, 2^nd^, or 3^rd ^grade of junior high school, we assumed their ages to be 13, 14, and 15 years old, respectively.

### 1) Gender differences

Of the 195 cases, 89 (45.6%) were male and 106 (54.4%) were female. The male: female ratio of 1: 1.19 demonstrated a slight predominance of females over males.

### 2) Age distribution

**Figure 1:**
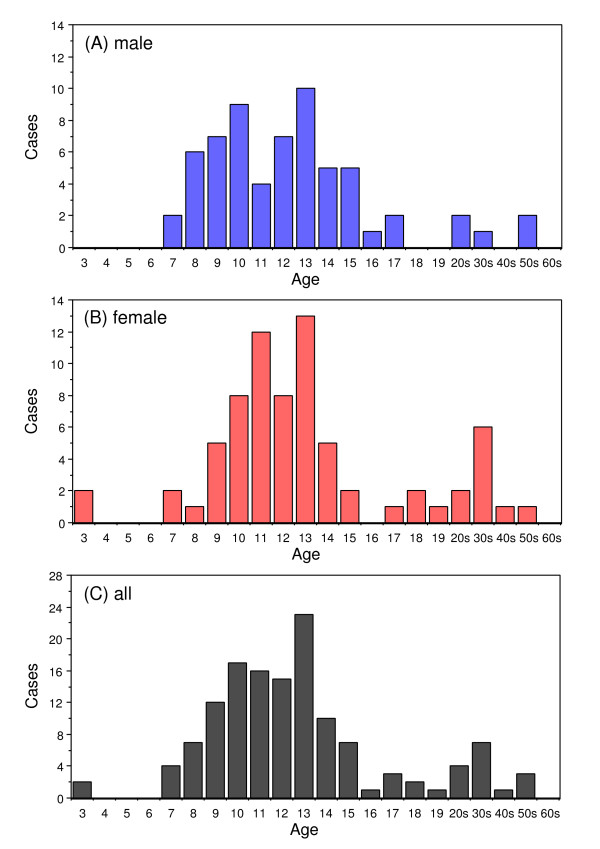
Age distribution of psychogenic fever patients by sex: Male (A), female (B) and both sexes (C).

In the type 1 papers, psychogenic fever patients ranged from 3 to 56 years old. The youngest case was a three-year-old girl who developed a 38°C fever each time she sat in a dentist's chair but had the fever return to within the normal range when she went home [39]. The oldest case was a fifty-six-year-old man with depression who had a persistent low-grade (37–38°C) fever in psychologically stressful situations [22]. The mean age at onset was 13.7 ± 8.6 years for males, 14.9 ± 9.3 years for females: 14.3 ± 9.0 for all cases. Age was not statistically different between males and females. The mode was 13 years for both sexes (Fig. 1). Patients between 11 and 15 years old were the most numerous: 52.6% of all cases (71). Patients between 6 and 10 years old were the second largest group: 40 cases (29.6%).

Among the type 2 papers, three reported on 10 or more cases [10,14,16]. All three papers were published by pediatricians and concluded that psychogenic fever is most frequently seen in 12–13 year-olds [10], 50% of psychogenic fever patients are 13 years old [14] and 72% of psychogenic fever patients are 12–14 years old [16]. These results were comparable to those of the type 1 papers.

### 3) The rate of psychogenic fever by department

Most reports on psychogenic fever were published by pediatricians (22 papers, 35.5%). Others were contributed by physicians specializing in psychosomatic medicine (19 papers), psychiatry (11 papers), internal medicine (2 papers), anesthesiology (2 papers), obstetrics/gynecology (1 paper), dentistry (1 paper), psychotherapy (3 papers) and education (1 paper). Examples of psychogenic fever in three departments are as follows.

### Pediatrics

*Hashira et al*. [24] reviewed fever of unknown origin (FUO) in children and reported that 23 (18.3%) out of 126 patients with FUO were finally diagnosed as psychogenic fever. The rate of psychogenic fever was 1.9–5.8% of all patients with functional psychosomatic diseases who visited the department of pediatrics outpatient clinic at Nagoya City Hospital in 1970, 1975, 1980, and 1985 [28].

### Psychiatry

Among outpatients who visited the department of child psychiatry at Hamamatsu Medical School between 1982 and 1985, the percentage of psychogenic fever patients was 5.2% [20].

### Obstetrics/gynecology

Among climacteric outpatients who visited an obstetrics/gynecology clinic complaining of low-grade fever, 8 out of 182 patients (4.4%) were diagnosed with psychogenic fever [54].

## Discussion

This study reviewed 195 cases of psychogenic fever, the largest number of cases that has ever been investigated and compiled in Japan. The results showed that the age of patients ranged from 3 to 56 years old, peaking at 13 years old, and that the male: female ratio of 1:1.19 showed a slight predominance of female cases over male cases. Most psychogenic fever patients visit pediatricians; however, such patients were seen in a wide variety of departments including psychosomatic medicine, psychiatry, and gynecology.

### Ages

Psychogenic fever patients were predominantly seen between 8–15 years-of-age, and especially at 13 years of age, in both sexes. This age range corresponds to the onset and duration of adolescence. Therefore, although the reasons for such an age distribution are unclear, autonomic and hormonal changes or psychological instability in adolescence may contribute to the high rates seen at these ages. Another possibility is that pediatricians are more aware of stress-induced hyperthermic responses because children have a higher body temperature than adults. For example, patients between 10–15 years sometimes exhibited temperatures of 39–41°C [3,4,7,11,32,33,35,38,43,48]. In contrast, most adult patients showed only 37–38°C [5,22,34,46,49,53,55,60-62]. Therefore, in the case of adult patients, physicians might pay little attention to hyperthermic responses, including those that are produced by psychological stress. Interestingly, there were only four cases reported in the 40–59 years-old range. However, considering that 4% of the low-grade fever of climacteric patients is psychogenic fever, there should be more patients in this age range. Some adult patients might be diagnosed with chronic fatigue syndrome (CFS) because CFS patients also have low-grade fever of unknown origin and physical symptoms exacerbated by stress. However, psychogenic fever is different from CFS [59] for the following reasons. The onset of psychogenic fever is not sudden. Psychogenic fever patients complain little of muscle weakness, myalgia, arthralgia, or chill. Cervical lymphadenopathy or infected pharynx is not observed. Furthermore, psychogenic fever is curable by stress reduction and conflict resolution via psychotropic drugs and psychotherapy.

### Gender differences

Several reports discussed gender differences in psychogenic fever. However, the results were conflicting: a predominance of males [16], females [20], or no difference [14]. The reasons for contradictory results may partly be due to the relatively small numbers of cases investigated (10–27 cases). The present study reviewed the largest number of cases ever published and revealed that the male: female ratio was 1: 1.19, with a slight predominance of female cases. It is possible that gender differences are related to the age of the patients. For example, 24 male and 18 female patients were under 11 years old, a male: female ratio of 1.33: 1. In contrast, 21 male and 33 female patients were between 11–13 years old, a male: female ratio of 1: 1.57.

Our findings of age and gender differences of psychogenic fever patients in Japan differ from those from the United States, where psychogenic fever is most likely to occur in young women, especially women from 20 to 40 years old [63-65].

### Prevalence

This study also revealed that psychogenic fever is not uncommon in the clinical setting and that patients with psychogenic fever are seen in a variety of departments. Psychogenic fever accounted for 18% of FUO in children and 2–6% of outpatients who visited psychosomatic pediatrics and child psychiatry. In spite of the high rates, the prevalence of psychogenic fever has yet to be investigated. Thus, further study is needed.

### Diagnosis

It is well known that psychological stress increases Tc in mammals [66]. For example, cage change or removal of mice that were housed in the same cage induced a 1.5–2.0°C increase in Tc in mice [67]. This phenomenon is called psychological stress-induced hyperthermia (PSH) in the field of basic science. This phenomenon is also observed in humans [for review, see [66]]. In most cases, however, the increased Tc rarely exceeds the upper limit of normal temperature and disabling physical symptoms are not observed. The reasons are still uncertain as to why psychogenic fever patients have high temperature caused by psychological stress and complain of numerous physical symptoms. In the clinical papers we reviewed, psychogenic fever refers to an acute or persistent increase in Tc above the normal limits (in most cases, 37°C). Animal studies have demonstrated that the mechanisms of PSH are different from those that cause inflammation-induced "fever" [66-68]. Therefore, although the term "psychogenic fever" is widely used, there is room for discussion of whether the term "fever" is appropriate.

## Conclusion

Psychogenic fever patients ranged from 3 to 56 years old, with the highest number of cases occurring at 13 years in both sexes. The male: female ratio of 1 : 1.19 suggests a slight predominance of female cases. Although Japanese psychogenic fever patients were mostly seen in the department of pediatrics, patients were also observed in a variety of departments, including psychosomatic medicine, psychiatry, and internal medicine.

## Competing interests

The author(s) declare that they have no competing interests.

## Authors' contributions

TO reviewed the papers and drafted the manuscript. KO reviewed the papers and analyzed the data. All authors read and approved the final manuscript.
